# The impact of pretransplant 25-hydroxy vitamin D deficiency on subsequent graft function: An observational study

**DOI:** 10.1186/1471-2369-13-22

**Published:** 2012-07-10

**Authors:** Hyunwook Kim, Shin-Wook Kang, Tae-Hyun Yoo, Myoung Soo Kim, Soon Il Kim, Yu Seun Kim, Kyu Hun Choi

**Affiliations:** 1 Department of Internal Medicine, Wonkwang University College of Medicine Sanbon Hospital, Gunpo-si, Kyunggi-do, Korea; 2 Department of Internal Medicine, Yonsei University College of Medicine, 134 Shinchon-dong, Seodaemun-gu, Seoul, 120-752, Korea; 3 Department of Surgery, Yonsei University College of Medicine, Seoul, Korea; 4 The Research Institute for Transplantation, Yonsei University College of Medicine, Seoul, Korea

## Abstract

**Background:**

In addition to its canonical role in musculoskeletal health, several reports have demonstrated that serum vitamin D level may influence kidney function. However, the effect of pretransplant serum vitamin D level on subsequent graft function has not been explored. Therefore, this study was undertaken to examine the effect of serum vitamin D level at the time of kidney transplantation (KT) on subsequent graft function.

**Methods:**

We analyzed 106 patients who underwent KT and for whom 25-hydroxy vitamin D (25-OHD) levels were measured during hospitalization prior to transplantation. We measured estimated glomerular filtration rates (eGFR) using the Modification of Diet in Renal Disease (MDRD) formula at baseline and at six-month intervals up to 36 months after KT.

**Results:**

38.7% of the patients were diagnosed with 25-OHD deficiency defined as less than 10 ng/mL. Recipient gender (female vs. male, odds ratio [OR] 3.30, 95% CI 1.33-8.21, *P* = 0.010), serum albumin level (per 1 mg/dl increase, OR 0.35, 95% CI 0.13-0.98, *P* = 0.047), and predominant renal replacement therapy modality before KT (*P* < 0.001) were found to be independent pretransplant risk factors for 25-OHD deficiency by multivariate logistic regression analysis. Subsequent repeated measures analysis of covariance revealed that 25-OHD level had the only significant main effect on eGFR during the 36-month follow-up period [*F* (1, 88) = 12.07, *P* = 0.001].

**Conclusions:**

Pretransplant 25-OHD deficiency was significantly associated with a lower post-transplant eGFR, suggesting that 25-OHD may play an important role in maintaining graft function after KT.

## Background

25-hydroxy vitamin D (25-OHD) is not only the stored form of vitamin D in the human body but also the predominant circulating form of vitamin D in the blood. Due to its long half-life and high concentration in plasma, the serum 25-OHD level is considered to be the best measure of vitamin D status. In addition to its classical role in maintenance of bone and neuromuscular health, several reports have demonstrated that poor vitamin D status is associated with a higher prevalence of hypertension, chronic heart failure, malignancy [1-3], and increased mortality not only in patients with chronic kidney disease not on dialysis, but also in incident hemodialysis patients [4,5]. Moreover, several clinical and experimental studies have reported a protective role for vitamins D against both diabetic and non-diabetic renal injuries [6-10].

Patients with kidney failure are at high risk for 25-OHD deficiency, most likely due to reduced exposure to sunlight resulting from inactivity and hyperpigmentation and, to some extent, reduced ingestion of foods that are natural sources of vitamin D [11]. Even though patients undergoing kidney transplantation (KT) are generally healthier than patients with end-stage kidney disease not eligible for KT, Sadlier et al*.* reported that only 12% of patients at the time of KT had a normal 25-OHD concentration (> 30 ng/mL), while 29% of patients had 25-OHD deficiency (< 10 ng/mL) [12]. To date, there has been little research exploring the consequences of this high prevalence of vitamin D deficiency at the time of KT.

We therefore undertook this study to examine which clinical parameters at the time of KT are associated with vitamin D deficiency and the effect of vitamin D status prior to KT on subsequent graft function in incident kidney transplant patients.

## Methods

### Study population

**Figure 1:**
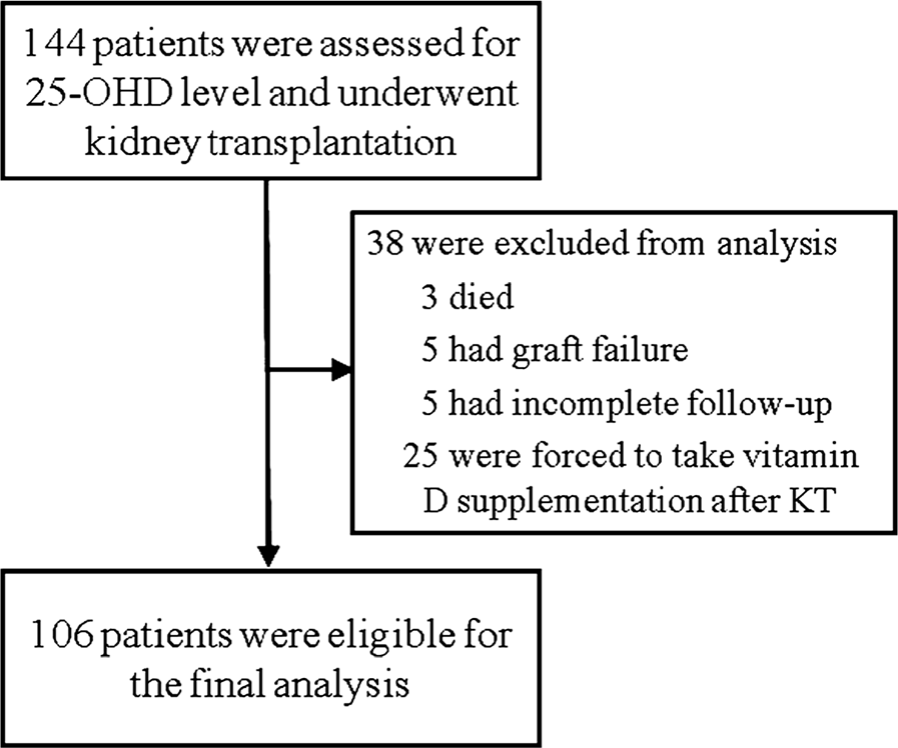
**Study profile.** All 106 patients selected from 144 patients who were screened for 25-OHD level were included in the final analysis.

One hundred forty-four patients who had been hospitalized for KT at Yonsei University Health System in Seoul, Korea (latitude: 37.5°N; annual average sunshine hours: 5.8 hours *per* day) between April 1, 2002 and June 30, 2004 were enrolled and had their 25-OHD level assessed. All participants were ethnically homogeneous Korean population. At enrollment, patients who were less than 16 years old, had active liver disease or liver cirrhosis, or had received prior KT were excluded. During follow-up period, patients who were forced to take vitamin D supplements after KT for compelling indications, such as pre-existing uremic osteodystrophy or post-transplant osteoporosis, or who did not have an equal number of observations (estimated renal function) made at fixed regular intervals were also excluded from the analysis without imputation of missing data to minimize estimation bias in statistics used in this study (details are below) [13]. Therefore, 106 participants, who maintained functioning grafts and were regularly assessed for graft function without missing data throughout the 36-month study period, were only included in the final analysis (Figure 1).

Baseline demographic and clinical characteristics and laboratory data including 25-OHD level were obtained during hospitalization prior to transplantation. For correcting serum total calcium for the effects of confounders, such as protein level or nutritional status, albumin-corrected calcium was calculated as previously described [14]. Predominant renal replacement therapy (RRT) during the pretransplant end-stage renal disease (ESRD) course was defined as the RRT modality for >50% of the ESRD period.

Of the screened 144 patients, 16 patients (11.1%) experienced acute rejection episodes. Diagnosis of all these rejection episodes was confirmed by biopsy. Of whom, 11 patients were diagnosed with acute cellular rejection and were treated with 1 g boluses of intravenous methyl prednisolone for 3 consecutive days or additional polyclonal/monoclonal antibody therapies for steroid-resistant cases (3 of 11 episodes), which enabled them to maintain graft patency and be included in the final analysis. In contrast, 5 patients were diagnosed with acute mixed humoral/cellular rejection and lost their grafts despite receiving intensive courses of immunosuppressive therapy and returned to dialysis (Figure 1).

These patients were prospectively evaluated for graft function at six-month intervals for 36 months following KT by estimated glomerular filtration rate (eGFR) using the Modification of Diet in Renal Disease (MDRD) equation. When analyzing serum 25-OHD concentrations in this population, 25-OHD deficiency was defined as a serum 25-OHD < 10 ng/mL because this level is associated with an increased risk of rickets in children and osteomalacia in adults [15]. This study was approved by the Institutional Review Board of Severance Hospital, Yonsei University College of Medicine (no. 4-2009-0382) and written informed consent was obtained from all subjects.

### Measurement of serum 25-OHD level

All specimens measured for 25-OHD were examined in the endocrinology laboratory at the Medical Research Center of Yonsei University Health System. 25-OHD level was evaluated using a Diasorin 25-OHD ^125^I radioimmunoassay kit [16], sensitivity ≤ 1.5 ng/mL; inter-assay coefficient of variation (CV) = 11.7% at 8.6 ng/mL and 8.6% at 33.0 ng/mL.

### Immunosuppressive regimens

During the study period, most patients were treated with a triple regimen of calcineurin inhibitor (cyclosporine or tacrolimus), purine synthesis inhibitor (azathioprine or mycophenolate mofetil), and prednisolone.

### Statistical analysis

Data analysis was performed using SPSS for Windows, version 12.0 (SPSS, Chicago, IL, USA). All results are presented as the mean ± standard error of the mean (SEM). The independent *t*-test was used to compare means and analysis of variance (ANOVA) with Bonferroni post-hoc test was used for multiple comparisons between groups. The Chi-square test was used to compare proportions between variables. Logistic regression analysis was used to estimate the odds ratios and to identify the independent pretransplant risk factors for 25-OHD deficiency; all variables at the *P* < 0.1 level in univariate analyses and recipient age at KT were included in the model.

To evaluate the impact of 25-OHD deficiency on changes in eGFR during the 36 months following KT, a repeated measures analysis of covariance (ANCOVA) was conducted with recipient gender, predominant RRT modality before KT, Biopsy-proven acute rejection episode, and group according to serum 25-OHD concentration (25-OHD deficiency vs. control) as between-subjects factors, the eGFR at each six-month intervals after KT as a within-subjects factor, and baseline serum albumin level as a covariate. Because our data violated sphericity assumptions, multivariate tests using Wilks’ lambda was used to analyze of the within-subjects effects instead of univariate tests. All main effects as well as their interactions were entered into the models. If there was a significant interaction effect, the Bonferroni correction was used for post hoc repeated measures ANCOVA. In all cases, a *P*-value less than 0.05 was considered significant.

## Results

### Demographic features

Demographic characteristics of the patients analyzed are listed in Table 1. The mean age was 40.2 ± 1.1 years (range: 16.8–60.6 years), and 31 participants (29.2%) were women. Forty-nine (46.2%) patients had received hemodialysis, and 22 (20.2%) had received peritoneal dialysis as a predominant RRT modality prior to transplantation. The mean duration of dialysis for these 71 patients was 16.8 ± 2.7 months. The remaining 35 subjects (33.0%) received pre-emptive transplants.

**Table 1:** Baseline characteristics of the 106 patients

25-OHD (ng/ml)	13.1 ± 0.6
Demographic characteristics	
Recipient age (years)	40.2 ± 1.1
Recipient gender (% women)	31 (29.2)
Active vitamin D supplementation before KT	17 (16.0)
Etiology of renal failure (%)	
Diabetes mellitus	10 (9.4)
Glomerulonephritis	32 (30.2)
Hypertension and others	64 (60.4)
Duration of dialysis (months)	16.8 ± 2.7
History of cardiovascular disease	8 (7.5)
Smoking	22 (20.8)
Body mass index (kg/m^2^)	22.8 ± 0.3
Predominant RRT modality before KT (%)	
Hemodialysis	49 (46.2)
Peritoneal dialysis	22 (20.8)
Pre-emptive transplantation	35 (33.0)
Transplant type	
Living-related donor	76 (71.7)
Living-unrelated donor	29 (27.4)
Deceased donor	1 (0.9)
Donor characteristic	
Donor age	35.1 ± 1.0
Donor gender (% women)	46 (43.4)
ABO mismatch (%)	15 (14.2)
Number of HLA mismatches	2.8 ± 0.1
Mismatch at A locus (%)	83 (78.3)
Mismatch at B locus (%)	90 (84.9)
Mismatch at DR locus (%)	87 (82.1)
Laboratory test results	
Albumin (g/dl)	3.8 ± 0.0
Calcium (mg/dl)	9.1 ± 0.1
Albumin-corrected calcium (mg/dl)	9.3 ± 0.1
Phosphorus (mg/dl)	5.8 ± 0.2
Alkaline phosphatase (IU/l)	68.6 ± 2.6
Parathyroid hormone (intact; pg/ml)	204.6 ± 19.9
Osteocalcin (ng/ml)	45.2 ± 4.0
Hemoglobin (g/l)	9.5 ± 0.2
Biopsy-proven acute rejection episode	11 (10.4)

Results are expressed as mean ± SEM or number (%).

KT, kidney transplantation; RRT, renal replacement therapy.

### Distribution of 25-OHD concentrations

**Figure 2:**
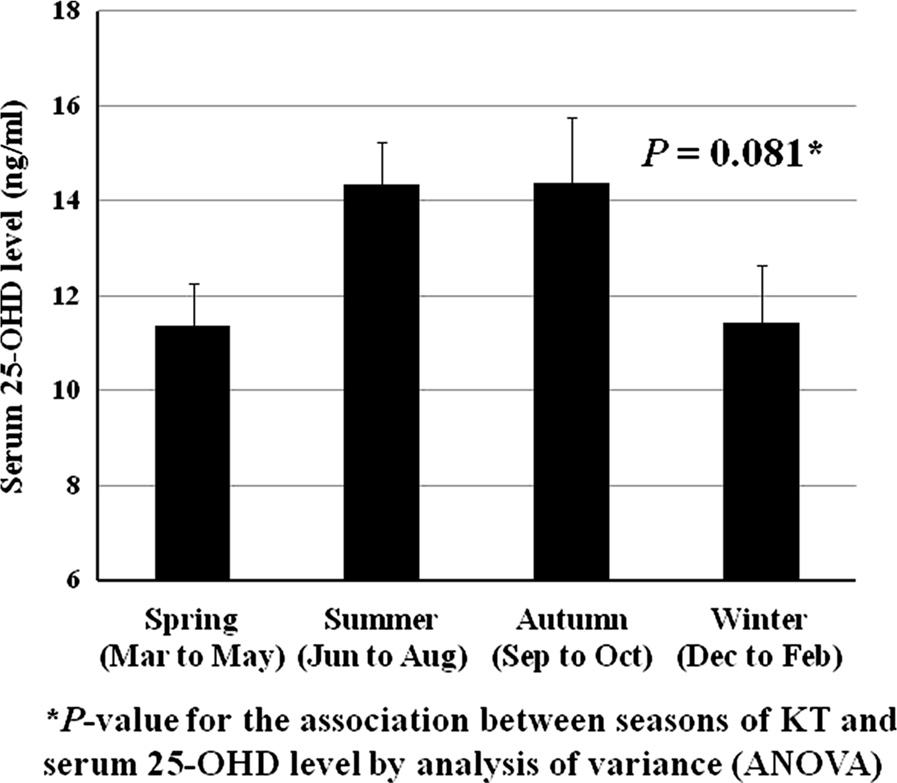
**Seasonal variations of pretransplant 25-OHD levels.** I bars represent the standard error. *P-value for the association between seasons of KT and serum 25-OHD level by analysis of variance (ANOVA).

The mean serum 25-OHD concentration was 13.1 ± 0.6 ng/mL; 41 participants (38.7%) had vitamin D deficiency defined as less than 10 ng/mL. Considering the latitude of Korea, it can be assumed that there is a seasonal variation in ultraviolet exposure, which is a major determinant of 25-OHD level. Therefore, we analyzed the seasonal variation of pretransplant 25-OHD levels. As shown in Figure 2, there was a trend that serum 25-OHD levels were higher in summer (14.3 ± 0.9 ng/mL)/autumn (14.4 ± 1.4 ng/mL) than in spring (11.4 ± 0.9 ng/mL)/winter (11.4 ± 1.2 ng/mL), but did not reach statistical significance both in ANOVA (*P* = 0.081) and post-hoc multiple comparison using Bonferroni test.

Characteristics of the patients according to serum 25-OHD level are shown in Table 2. Results showed that the patients divided into two groups according to 25-OHD level were similar with regard to donor age and gender, percentage of living and deceased donors, the number and locus of HLA mismatch, percentage of ABO mismatch, etiology of renal failure, and duration of dialysis. However, the patients with 25-OHD deficiency at KT were more likely to be female (*P* = 0.002), had received maintenance peritoneal dialysis as a predominant RRT modality prior to KT (*P* < 0.001), experienced more frequent biopsy-proven acute rejection episodes (*P* = 0.003), and had lower serum levels of hemoglobin (*P* = 0.028) and albumin (*P* < 0.001). Serum calcium level was also lower in the patients with 25-OHD deficiency (*P* = 0.004), but lost significance after correcting for serum albumin level (*P* = 0.121).

**Table 2:** Baseline characteristics of patients according to serum 25-OHD levels

	Without 25-OHD deficiency (n = 65)	With 25-OHD deficiency (n = 41)	*P*- values
25-OHD (ng/ml)	16.7 ± 0.6	7.3 ± 0.3	< 0.001
Demographic characteristics			
Recipient age (years)	40.5 ± 1.3	39.9 ± 1.8	0.793
Recipient gender (% women)	12 (18.5)	19 (46.3)	0.002
Active vitamin D supplementation before KT	13 (20.0)	4 (9.8)	0.186
Etiology of renal failure (%)			
Diabetes mellitus	5 (7.7)	5 (12.2)	0.440
Glomerulonephritis	17 (26.2)	15 (36.6)	0.255
Hypertension and others	43 (66.2)	21 (51.2)	0.126
Duration on dialysis (months)	19.1 ± 3.7	13.3 ± 3.9	0.309
History of cardiovascular disease	5 (7.7)	3 (7.3)	0.943
Smoking	15 (23.1)	7 (17.1)	0.458
Body mass index (kg/m^2^)	23.2 ± 0.4	22.2 ± 0.4	0.102
Predominant RRT modality before KT (%)			
Hemodialysis	42 (64.6)	7 (17.1)	< 0.001
Peritoneal dialysis	6 (9.2)	16 (39.0)	< 0.001
Pre-emptive transplantation	17 (26.2)	18 (43.9)	0.058
Transplant type			
Living-related donor	44 (67.7)	32 (78.0)	0.249
Living-unrelated donor	20 (30.8)	9 (22.0)	0.321
Deceased donor	1 (1.5)	0 (0.0)	1.000
Donor characteristic			
Donor age	35.2 ± 1.4	35.0 ± 1.5	0.922
Donor gender (% women)	36 (56.3)	23 (56.1)	0.988
ABO mismatch (%)	12 (18.8)	3 (7.3)	0.153
Number of HLA mismatches	2.9 ± 0.2	2.8 ± 0.2	0.753
Mismatch at A locus (%)	49 (76.6)	34 (82.9)	0.434
Mismatch at B locus (%)	55 (85.9)	35 (85.4)	0.935
Mismatch at DR locus (%)	52 (81.3)	35 (95.4)	0.585
Biopsy-proven acute rejection episode	2 (3.1)	9 (22.0)	0.003
Laboratory test results			
Albumin (g/dl)	4.0 ± 0.1	3.6 ± 0.1	< 0.001
Calcium (mg/dl)	9.4 ± 0.1	8.7 ± 0.2	0.004
Albumin-corrected calcium (mg/dl)	9.4 ± 0.1	9.1 ± 0.2	0.121
Phosphorus (mg/dl)	6.0 ± 0.3	5.5 ± 0.2	0.169
Alkaline phosphatase (IU/l)	68.9 ± 3.3	68.0 ± 4.4	0.864
Parathyroid hormone (intact; pg/ml)	205.4 ± 26.9	203.2 ± 29.2	0.957
Osteocalcin (ng/ml)	47.7 ± 5.4	41.2 ± 5.5	0.429
Hemoglobin (g/l)	9.8 ± 0.2	9.0 ± 0.3	0.028

Results are expressed as mean ± SEM or number (%).

KT, kidney transplantation; RRT, renal replacement therapy.

Among the pretransplant parameters, an adjusted multivariate logistic regression model showed that female gender was independently associated with 25-OHD deficiency (odds ratio [OR] 3.30, 95% CI 1.33–8.21, *P* = 0.010), and increased serum albumin level was associated with lower odds of 25-OHD deficiency (OR 0.35, 95% CI 0.13–0.98, *P* = 0.047). In addition, predominant RRT modality before KT was also associated with 25-OHD deficiency (*P* < 0.001) (Table 3).

**Table 3:** Independent predictors for 25-OHD deficiency using multivariate logistic regression model^*^

Pretransplant parameters	B	S.E.	Wald	*df*	*P*- values	OR	95% CI
Female sex vs. male)	1.19	0.47	6.60	1	0.010	3.30	1.33-8.21
Serum albumin (per 1 mg/dl increase)	−1.04	0.52	3.96	1	0.047		0.13-0.98
Predominant RRT modality before KT			13.66	2	< 0.001	0.35	
HD (vs. PD)	−2.31	0.65	12.64	1	< 0.001	0.10	0.03-0.36
Preemptive KT (vs. PD)	−0.96	0.60	2.56	1	0.110	0.38	0.12-1.24

^*^adjusted for recipient age and baseline serum hemoglobin level.

25-OHD, 25-hydroxy vitamin D; RRT, renal replacement therapy; KT, kidney transplantation; HD, hemodialysis; PD, peritoneal dialysis.

### Changes in eGFR

**Figure 3:**
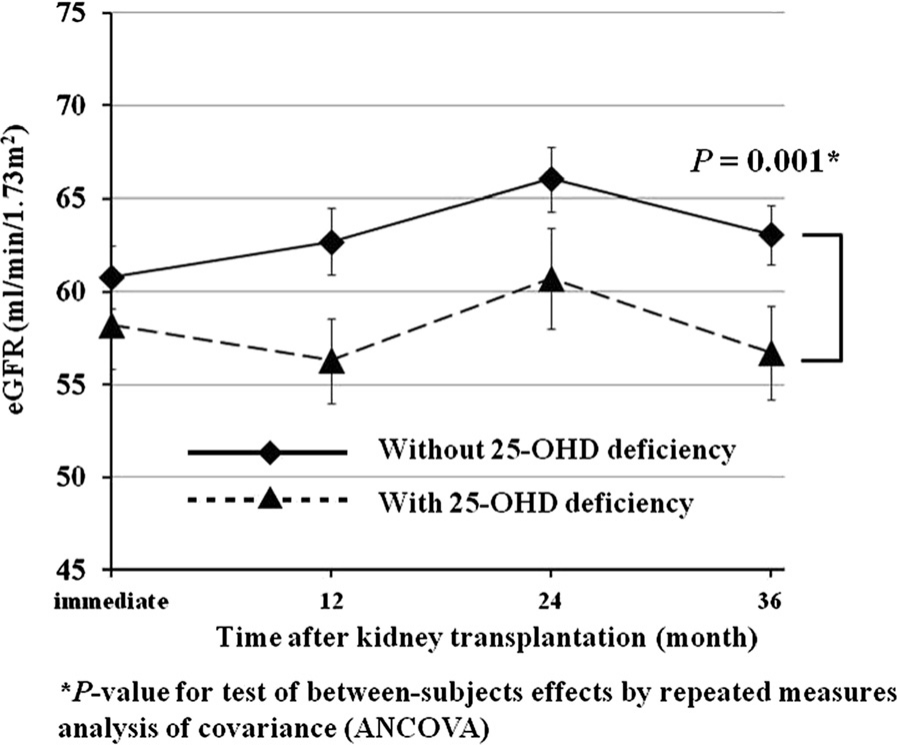
**Changes in eGFRs between patients with and without 25-OHD deficiency from the immediate postoperative period (20 days after kidney transplantation) to 36 months after kidney transplantation.** I bars represent the standard error. *P-value for test of between-subjects effects by repeated measures analysis of covariance (ANCOVA).

A repeated measures ANCOVA was performed to explore whether pretransplant 25-OHD level had an impact on changes in eGFR during the 36-month follow-up period as a main between-subjects factor. We also entered recipient gender and predominant RRT modality before KT as between-subjects factors, and serum albumin level as a covariate into the model because they were potential pretransplant contributors to differences in 25-OHD level (Table 3). Since an acute rejection episode is an established precedent for chronic graft dysfunction [17] and the incidence of posttransplant biopsy-proven acute rejection was significantly different according to 25-OHD level (Table 2), a biopsy-proven acute rejection episode was also included as a between-subjects factor. The within-subjects factor was represented by the eGFR at each six-month interval after KT. Upon multivariate test of within-subjects effect, there was no significant main effect for eGFR at each 6-month interval after KT [Wilks’ lambda = 0.92, F(6, 83) = 1.39, *P* = 0.228], whereas eGFR at each 6-month interval after KT by group (25-OHD deficiency vs. control) interaction was significant [Wilks’ lambda = 0.84, F(6, 83) = 3.56, *P* = 0.003]. This indicates that, while overall graft function did not change significantly throughout the 36 months after KT, the patterns of changes in graft function were not consistent and depend on 25-OHD level. Subsequent analysis of between-subjects effects revealed that only the main effect of group according to 25-OHD level (25-OHD deficiency vs. control) was significant, and none of the interactions associated with group according to 25-OHD level (25-OHD deficiency vs. control) were significant [F(1, 88) = 12.07, *P* = 0.001] (Figure 3) (Table 4). While the main effect of biopsy-proven acute rejection episode was not significant, there were significant interactions of Biopsy-proven acute rejection episode with recipient gender [F(1, 88) = 6.78, *P* = 0.011] and predominant RRT modality before KT [F(1, 111) = 3.84, *P* = 0.025]. These results suggest that an acute rejection episode affects post-KT graft function differently according to recipient gender and predominant RRT modality before KT. Further, post hoc repeated measures ANCOVA analyses with the Bonferroni corrections revealed that, after an biopsy-proven acute rejection episode, males experienced a persistently lower level of graft function [F(1, 73) = 7.57, *P* = 0.007] but females did not [F(1, 29) = 0.65, *P* = 0.426], and only the graft function of patients receiving pre-emptive KT was affected adversely by a biopsy-proven acute rejection episode [F(1, 33) = 5.86, *P* = 0.020] but patients receiving HD [F(1, 47) = 1.80, *P* = 0.185] or PD [F(1, 20) = 0.28, *P* = 0.605] as predominant RRT modality before KT were not adversely affected.

**Table 4:** Test of between-subject effects on change of eGFR using repeated measures ANCOVA^*^

Between-subjects factors	*df*	F	*P*-values
Recipient gender	1	3.03	0.085
Predominant RRT modality before KT	2	2.50	0.087
Biopsy-proven acute rejection episode	1	1.83	0.179
25-OHD deficiency	1	12.07	0.001

^*^Analysis was conducted with all listed between-subjects factors and baseline serum albumin level as covariate entered into models.

RRT, renal replacement therapy; KT, kidney transplantation; 25-OHD, 25-hydroxy vitamin D.

## Discussion

Poor vitamin D status, defined by low level of serum 25-OHD concentration, has been reported to be associated with both renal dysfunction and nephrotic syndrome [18]. In addition, 25-OHD level is positively correlated with eGFRs [19]. This correlation can in part be explained by certain conditions that are more prevalent in the dialysis population, including uremic inflammatory status, co-morbidities, dark skin color, decreased sun exposure, poor nutritional status, and decreased vitamin D binding protein.

Our study showed that 38.7% of the analyzed patients suffered from 25-OHD deficiency, with a mean value of 13.1 ± 0.6 ng/mL. Men had significantly higher levels of 25-OHD than did women, consistent with previous studies conducted in both the general population [20] and in patients with chronic kidney disease [19]. We also discovered that low serum albumin level was associated with low 25-OHD level, which might be explained not only by malnutrition or uremic inflammation but also by the positive correlation between serum albumin and vitamin D binding protein levels [21].

Patients with 25-OHD deficiency were more likely to be receiving peritoneal dialysis than hemodialysis prior to KT. This can be explained by a substantial loss of vitamin D binding protein through the peritoneal membrane [22] or direct loss of 25-OHD via peritoneal membrane into PD fluid [23], which is unlikely in conventional hemodialysis due to a general inability to transport middle-sized proteins.

A principal finding of our prospective study was that 25-OHD level at the time of KT had a substantial effect on subsequent graft function. Several experimental studies demonstrated that the active form of vitamin D, 1,25-dihydroxyvitamin D [1,25-(OH)_2_D] has a potentially beneficial effect on the progression of renal disease. Vitamin D/vitamin D analog therapy has been shown to decrease glomerulosclerosis by suppressing the actions of TGF-β [6], albuminuria, podocyte hypertrophy [8], mesangial cell proliferation [24], and activation of the renin-angiotensin system [9]. In addition, other possible renoprotective roles more specifically related to transplantation have also been noted. There was a tendency toward fewer acute rejection episodes in 1,25-(OH)_2_D-treated kidney transplant cohort [25]. Consistent with this finding, our study showed that 25-OHD deficiency was associated with a significantly higher incidence of biopsy-proven acute rejection (*P* = 0.003, Table 2). Several other immunomodulatory mechanisms potentially influencing graft function have also been explored, including attenuation of the activities of CD4^+^/CD8^+^ T-cells [26,27], B-cells [28], and dendritic cells [29] as well as alteration of TGFβ-1 and matrix-regulating molecules [30]. There is little conversion of 25-OHD to 1,25-(OH)_2_D due to lack of 1-α hydroxylase in patients with kidney failure; however, after KT, 1,25-(OH)_2_D levels can be restored by allograft [31].

Taken together, these facts imply that a suboptimal level of 25-OHD, as a substrate for 1,25-(OH)_2_D, at the time of KT might have an adverse effect on subsequent graft function from as early as 20 days (Figure 3) to several months after KT with its long half-life (up to 25–30 days) [32]. Additionally, it is well established that early graft function, under the possible influence of 25-OHD level at the time of KT, is the most relevant predictor for long-term graft function [33]. Furthermore, we cannot completely exclude the possibility that 25-OHD has another independent role in maintaining graft function not mediated by 1,25-(OH)_2_D.

This study had some limitations but also points of relevance. First, serum 25-OHD level was measured only once at baseline. But, Since posttransplant 25-OHD supplementation is the only proven intervention to change 25-OHD level in renal transplant patients [34], we excluded all the patients taking post-KT vitamin D supplements from the analysis. Therefore, we can cautiously expect that initial differences in 25-OHD might be maintained during the study period. Second, only serum 25-OHD level was measured, but not 1,25-(OH)_2_D level because only serum 25-OHD level is used as a standard to define vitamin D deficiency. In addition, a positive correlation between 25-OHD and 1,25-(OH)_2_D levels in transplant subjects with mild renal impairment suggests that the serum level of 25-OHD can be used as an estimate of 1,25-(OH)_2_D level [35]. Third, instead of imputation, we excluded the data of all the patients who did not have complete details of graft function at fixed intervals according to protocol. Excluded patients were those who experienced premature graft failure, were dead, or had even 1 missing data of graft function at fixed intervals during the study period. This process was required and helps to minimize the estimation bias in repeated measures ANCOVA, but meanwhile also might introduce sample bias since the subjects with complete data may not be representative of the entire transplant population. However, more than 90% of the participants, who were initially screened, had complete data of graft function at fixed intervals. Hence, such sample bias is expected to be relatively small.

## Conclusions

In conclusion, we found that 25-OHD deficiency at the time of KT had a significant adverse effect on graft function during the first 36 months after KT. Although the exact mechanism remains to be elucidated, vitamin D metabolites not only contribute to canonical musculoskeletal health but may also play an important role in the health of other organ systems, including maintenance of early graft function. Further studies are needed to identify an effective and safe method for maintaining adequate 25-OHD levels in dialysis patients listed for KT and to clarify the effect of vitamin D supplementation in vitamin D deficient KT patients on the post-transplant outcomes, which is expected to be addressed in the ongoing randomized controlled trial by Thiem et al. (ClinicalTrial.gov NCT00752401) [36].

## Competing interests

The authors declare that they have no competing interests.

## Author’s contributions

The authors’ responsibilities were as follows: HK designed and conducted the statistical analysis and wrote the manuscript; SWK and THY edited and made critical revision to the manuscript; MSK organized the data collection and helped interpret data; SIK and YSK collected data and contributed to study design; KHC organized the data collection and edited the manuscript. All authors approved the final version of the manuscript.

## Pre-publication history

The pre-publication history for this paper can be accessed here:

http://www.biomedcentral.com/1471-2369/13/22/prepub
